# The chemokine receptor CCR7 mediates corneal antigen-presenting cell trafficking

**Published:** 2007-04-27

**Authors:** Yiping Jin, Linling Shen, Eva-Marie Chong, Pedram Hamrah, Qiang Zhang, Lu Chen, M. Reza Dana

**Affiliations:** Schepens Eye Research Institute and the Massachusetts Eye & Ear Infirmary, Department of Ophthalmology, Harvard Medical School, Boston, MA

## Abstract

**Purpose:**

Trafficking of corneal antigen-presenting cells (APC) to draining lymph nodes (LN) is critical in triggering immune responses. However, very little is known about the molecular regulation of this pathway. We investigated the expression and function of the chemokine receptor CCR7 in mediating corneal APC migration in inflammation.

**Methods:**

Expression of CCR7 and its ligands, CCL21 and CCL19, in the normal and inflamed corneas was analyzed by RT-PCR and immunofluorescence staining. The phenotype of CCR7-expressing cells was identified by double-staining with different cell surface markers. To trace the trafficking of APC to draining LN, we injected corneal grafts with Alexa488-conjugated ovalbumin (OVA) and transplanted to syngeneic recipients. CCR7 expression on the Alexa488-conjugated OVA^+^ cells in the ipsilateral draining LN was analyzed by flow cytometry. To determine the functional role of CCR7, we injected anti-CCL21 neutralizing antibody subconjunctivally after corneal transplantation and analyzed changes in numbers of OVA^+^ cells in the draining LN. Each experiment was repeated at least three times.

**Results:**

Both CCR7 and its ligand CCL21 were significantly upregulated in inflamed corneas as measured by RT-PCR and immunofluorescence staining. CCR7^+^ cells were detected especially in the corneal periphery near LYVE-1^+^ lymphatic vessels. CCR7^+^ cells were universally CD11b^+^CD11c^+^, and a majority were major histocompatibility complex class II positive, suggesting a monocytic dendritic cell lineage and a relative state of maturation. Forty-eight h after syngeneic transplantation with OVA-loaded grafts, CCR7 expression was detected on the OVA^+^ cells in both the host corneal beds and the draining LN. Local administration of anti-CCL21 led to a significant suppression in the flow of OVA^+^CD11c^+^ cells to the draining LN.

**Conclusions:**

These data suggest that in inflammation, APC expressing CCR7 on their cell surface interact with CCL21 to facilitate their migration from the cornea to draining LN via afferent lymphatics.

## Introduction

During the past decade, peripheral tissue dendritic cells (DC) have been credited as the principal antigen-presenting cells (APC) in activating naïve T cells within secondary lymphoid tissues (e.g., lymph nodes [LN]) [1]. As such, DC are critical in immune surveillance in infectious diseases, cancer, transplantation, and allergy. Recent data from our laboratory have revealed that mature DC in the inflamed cornea, including both resident cells and those recruited from the vascularized areas around the cornea, including the limbus and conjunctival lymphatics, traffic to draining LN through the afferent lymphatics. Mature DC that express high levels of major histocompatibility complex (MHC) class II and B7 (CD80/CD86) costimulatory molecules can thus stimulate naïve T cells in the draining LN to induce immunogenic inflammation [2-5]. Disruption of this eye-LN axis (e.g., through surgical cervical and submandibular lymphadenectomy) has been shown to lead to both complete abrogation of host allosensitization and universal and indefinite allograft survival [6]. Therefore, determining the regulatory mechanisms of DC trafficking is a key issue in corneal immunology. Recently, we have been interested in using molecular strategies to nonsurgically sever the APC-lymphatic access, an approach we have termed "molecular lymphadenectomy".

Our previous work has shown that signaling through vascular endothelial growth factor receptor-3 (VEGFR-3) is critical for DC access to lymphatics, and that selective blockade of this can impair DC flow to draining LN and induction of alloimmunity [7,8]. However, VEGFR-3-based interventions have effects beyond APC trafficking: Sprouting blood and lymphatic vessels express VEGFR-3, and blockade of VEGFR-3 can eventually also alter hemangiogenic and lymphangiogenic responses [9]. Additionally, corneal epithelial VEGFR-3 has recently been shown to be an important "sink" mechanism for VEGF-C/D, suppressing their ligation of VEGFR-2, and hence angiogenesis [10]. Therefore, VEGFR-3 targeting is not wholly specific to APC trafficking. Thus, we have continued our search for other molecular mechanisms involved in regulation of DC trafficking.

CC chemokine receptor 7 (CCR7) is a receptor thought to be critical for the colocalization of mature DC and T cells in the local draining LN in several tissues [11-18]. Both CCR7 ligands, CCL19 (also known as macrophage inflammatory protein 3-β, MIP-3β), and CCL21 (also known as secondary lymphoid tissue chemokine, SLC), are expressed in the T-cell zones of secondary lymphoid organs. In addition, CCL21 is expressed by endothelial cells in lymphatic vessels and high endothelial venules [19,20]. Therefore, CCR7-mediated DC migration, guided by CCL19 and CCL21, results in accumulation of mature DC in the afferent lymphatics and the T-cell areas of draining LN. Studies in CCR7-deficient mice have revealed a marked defect in DC migration to LN and impaired primary immune responses [11,18]. However, the expression and function of CCR7 and their ligands in the inflamed cornea have not been reported to date. We therefore hypothesized that CCR7 and its ligands are essential for DC migration from the cornea to the draining LN.

The specific aim of this study was to examine the expression of CCR7 and its ligands in the inflamed cornea, and determine their effect on DC trafficking from the inflamed cornea to the draining LN using an OVA-loaded corneal graft model. The results demonstrated that migration of DC is facilitated by the interaction of the CCR7 expression on their surface with CCL21 secreted by the lymphatic vessels.

## Methods

### Animals

Six- to eight-week-old male BALB/c (Taconic Farms, Germantown, NY) mice were used in all experiments. The animals were anesthetized with a Katamine (120 mg/Kg BW) and Xylazine (20 mg/Kg BW) mixture before all surgical procedures. Carbon dioxide inhalation was applied to euthanize the animal before we harvested cornea and LN. All experimental protocols were approved by the Schepens Eye Research Institute Animal Care and Use Committee, and all animals were treated according to the Association for Research in Vision and Ophthalmology statement for the Use of Animals in Ophthalmic and Vision Research.

### Suture-induced inflamed cornea

Three interrupted sutures (11-0 nylon, Sharpoint; Vanguard, Houston, TX) were placed intrastromally with two stromal incursions extending over 120° of the corneal circumference. This procedure has been demonstrated to induce inflammatory corneal neovascularization, also associated with significant lymphangiogenesis [21]. After 10 days, when all corneas developed extensive neovascularization, all sutures were removed. Then corneas were carefully dissected by Vannas scissors around limbus for RNA isolation and immunohistochemical studies, to ensure that the conjunctival and iris tissues were not included.

### RNA isolation and reverse transcriptase-PCR

Corneas were harvested from the normal and inflamed mouse groups. Five corneas were involved in each group. To further dissect corneal epithelial and stromal-endothelial layers, another 5 corneas from inflamed mice were used. Each experiment was repeated three times. To extract mRNA from corneal epithelial and stroma-endothelial layers separately, five intact corneas were placed in 30 μl of RNA stabilization reagent (RNAlater, Qiagen, Valencia, CA) and then incubated in 250 μl of 20 mM EDTA (pH 7.4) at 37 °C for 30 min. Before mRNA isolation, the epithelial layers were peeled away from the stroma-endothelial layers. A combined method for total RNA isolation was employed, using Trizol (Invitrogen Corp., Carlsbad, CA) for tissue homogenization on ice, precipitation of RNA in the aqueous phase using 70% ethanol, followed by subsequent extraction and purification using RNeasy MinElute Spin Columns (Qiagen). Reverse transcription of total RNA was conducted using oligo(dT)15 primer and Sensiscript Reverse Transcriptase (Qiagen, Hilden, Germany). PCR was conducted using primer pairs for CCR7 (sense GAG GAA AAG GAT GTC TGC CAC G, antisense GGC TCT CCT TGT CAT TTT CCA G, 284 bp), CCL21 (sense CCA AGT TTA GGC TGT CCC ATC, antisense GGG CTA CTG GGC TAT CCT CT, 257 bp), CCL19 (sense CCT TCC GCT ACC TTC TTA ATG, antisense CTT CTG GTC CTT GGT TTC CTG, 229 bp), CXCR3 (sense CGC AAC TGT GGT CGA GAA AGC, antisense CAC AGG GAT GGC TGA GTT CTA C, 525 bp) and GAPDH (sense GAA GGG CAT CTT GGG CTA CAC, antisense GCA GCG AAC TTT ATT GAT GGT ATT, 373 bp). The PCR conditions were 35 cycles at 95 °C for 30 s, 58 °C for 30 s, and 72 °C for 1 min, followed by final extension at 72 °C for 10 min. The size of PCR products was determined by agarose gel electrophoresis.

### Immunohistochemical studies

Full thickness corneal tissue or 8 μm frozen sections were fixed in 4% paraformaldehyde for 30 min at 4 °C or acetone for 15 min at room temperature (RT; n=5 per group per experiment, repeated three times). To prevent nonspecific staining, anti-FcR mAb (CD16/CD31, FcγIII/II receptor) and streptavidin/biotin blocking solutions (Vector Laboratories, Burlingame, CA) were used to block sections before they were stained with primary antibodies or isotype-matched control antibodies at 4 °C overnight. Thereafter, the tissues were incubated with secondary antibodies at RT. For visualization of CCR7 staining, tyramide amplification was used according to the manufacturer's instructions (PerkinElmer Life Sciences, Boston, MA). Each step was followed by three thorough washings in PBS for 5-10 min. Finally, the samples were covered with mounting medium (Vector Laboratories) and analyzed by confocal laser scanning microscope (TCS 4D; Leica, Heidelberg, Germany) or epifluorescence microscope (Eclipse E800; Nikon, Tokyo, Japan). The following antibodies were used: FITC-conjugated mouse antimouse Iab (MHC class II), FITC-conjugated rat antimouse CD11b (a marker for granulocytes, monocytes, and macrophages), purified hamster antimouse CD11c (DC marker), biotin-conjugated rat antimouse CCR7 (eBioscience, San Diego, CA), biotin-conjugated goat antimouse CCL21 (R&D Systems, Minneapolis, MN), biotin-conjugated goat antimouse CCL19 (R&D Systems), and purified rabbit antimouse LYVE-1 (Abcam, Cambridge, MA). The secondary antibodies were Cy5-conjugated goat antihamster IgG (Jackson ImmunoResearch, West Grove, PA), Cy3-conjugated antibiotin IgG (Jackson ImmunoResearch), and FITC-conjugated donkey antirabbit IgG (Santa Cruz Biotechnology, Santa Cruz, CA). Isotype controls included FITC-conjugated mouse IgG2a, FITC-conjugated rat IgG2b, biotin-conjugated rat IgG2a, biotin-conjugated goat IgG (Santa Cruz Biotechnology), and purified rabbit IgG (Santa Cruz Biotechnology). Except where noted, primary antibodies and isotype-matched were purchased from BD Pharmingen (San Diego, CA).

### Syngeneic transplantation with ovalbumin-loaded corneal tissue

Fluorescently labeled antigens are useful for detecting the migration of macrophages and DC in the eye [22,23]. In our study, we injected Alexa488-labeled OVA into corneal grafts to track their migration (n=6 per experiment, repeated three times). Intrastromal injection with 2 μl of 2 μg OVA-Alexa488 (Molecular Probes, Eugene, OR) dissolved in PBS, or PBS alone, was performed in BALB/c mice through the limbus toward the central cornea, using a previously described technique [24,25]. Briefly, a small tunnel from the corneal epithelium to the anterior stroma was created using a 33-gauge needle (Hamilton Company, Reno, NV). Another 33-gauge needle attached to a 10 μl Hamilton syringe was passed through the tunnel into the stroma for the injection. After 24 h, these OVA-loaded grafts were placed onto neovascularized BALB/c host corneal beds. Our experiments employed a method of orthotopic corneal transplantation described in reference [26]. Briefly, a 2 mm corneal button was excised from the donor animal and grafted onto a 1.5 mm recipient corneal bed. Eight 11-0 interrupted nylon sutures were placed to secure the graft. Syngeneic corneal grafts never undergo immune rejection.

### Ocular administration of anti-CCL21

BALB/c mice were randomized to receive either anti-CCL21 (R&D systems) or isotype control goat IgG (Jackson ImmunoResearch) by subconjunctival injection in a masked fashion (n=3 per group per experiment, repeated three times). The antibodies were administered at a dose of 10 μg/10 μl per mouse on day -1, 0, 1 of corneal transplantation [12,27].

### Flow cytometry

Cells were harvested and pooled from ipsilateral submandibular LN of BALB/c recipients (n=3 per group per experiment, repeated three times) at 48 h posttransplantation. Upon blockade by anti-FcR mAb, cells were labeled with PE-conjugated anti-CD11c (BD Pharmingen) and quantified. Staining for CCR7 was done with a chimeric CCL19-Fc fusion protein (eBioscience), with PE-conjugated antihuman IgG (Fc-specific, eBioscience). Cells were subsequently washed and analyzed using an Epics XL flow cytometer (Beckman Coulter, Fullerton, CA). The percentage of cell subpopulations was quantified by three measurements, and the average was recorded. The percentage of OVA^+^ CD11c^+^ cells in the total cells of the draining LN was calculated with respect to isotype control staining. Mean values for each group (n=3) were compared via a two-tailed Student's t-test (GraphPad Prism 4.0c, San Diego, CA). A p-value <0.05 was considered significant.

## Results

### CCR7 is expressed in the inflamed cornea

**Figure 1:**
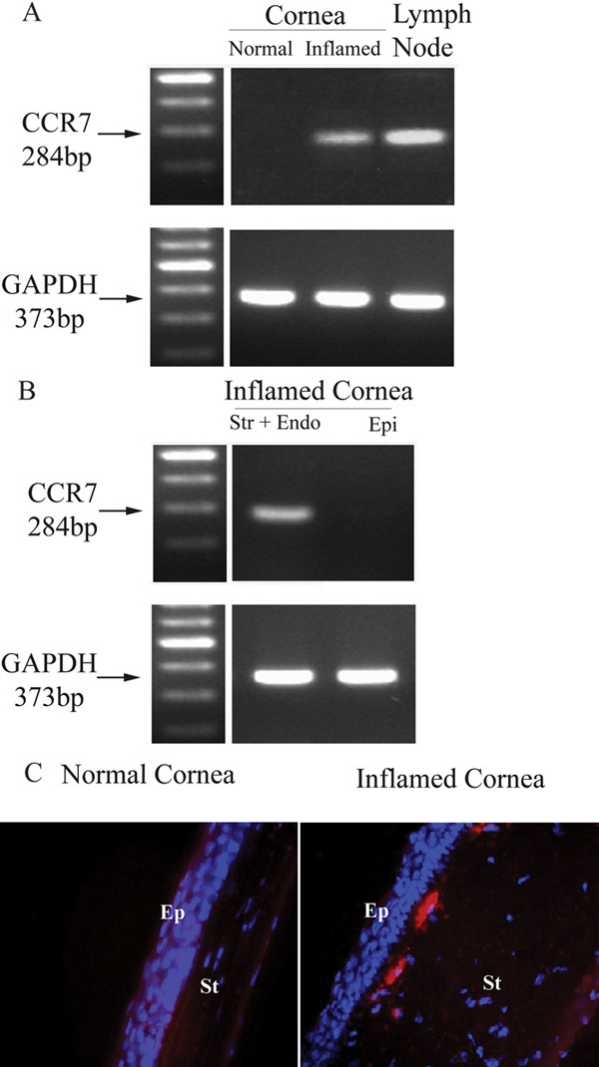
CCR7 is expressed in the inflamed corneal stroma. CCR7 expression in the cornea was first tested by RT-PCR. Lymph nodes served as a positive control. CCR7 mRNA expression was only detected in the inflamed cornea and not the normal one (**A**), and further identified in the stroma-endothelial layer (**B**: str indicates the stromal layer and endo designates the endothelia layer). CCR7 expression was displayed in red and the nuclear stain DAPI in blue on the cross-sections of the inflamed cornea (**C**). In the panels, Ep indicates corneal epithelium and St designates corneal stroma. Epifluorescence microscopy, original magnification: X40.

We initially investigated the expression of CCR7 in both the inflamed and the normal cornea by RT-PCR. CCR7 mRNA was detected in the inflamed cornea, but was absent in the normal cornea (Figure 1A). Further examination of the inflamed cornea revealed CCR7 mRNA in the stroma-endothelial layer, but not the epithelial layer (Figure 1B). Consistently, CCR7^+^ cells were shown by immunohistochemistry to localize only in the inflamed anterior corneal stroma (Figure 1C).

### CCR7^+^ cells in the inflamed corneal stroma represent bone marrow-derived dendritic cells

**Figure 2:**
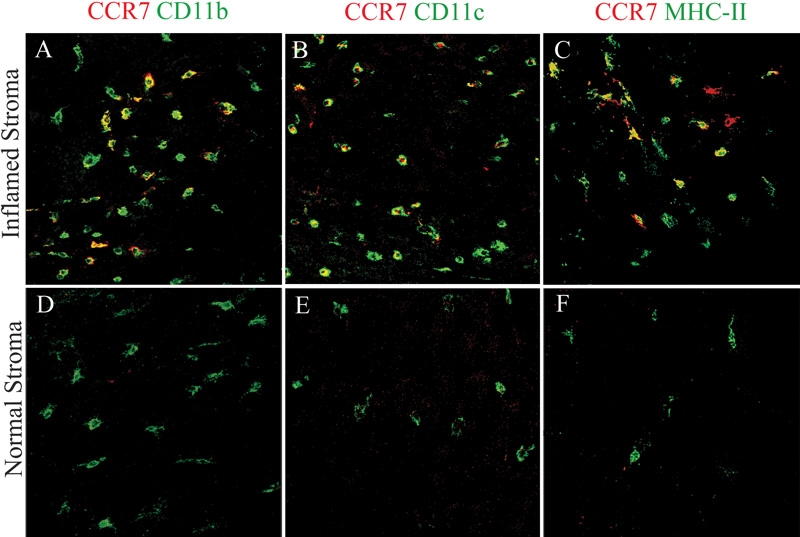
CCR7 was expressed by CD11b^+^ and CD11c^+^ cells in inflamed corneal stroma. CD11b (left), CD11b (left), CD11c (middle), and major histocompatibility complex (MHC) class II (right) were displayed in green in the normal and inflamed whole-mounted stromas. CCR7^+^ cells (red) were only detected in the inflamed (top panels), not the normal (bottom panels) corneal stromas. These cells were costained with CD11b (yellow; **A**) and CD11c (yellow; **B**). A majority of CCR7^+^ cells also co-expressed MHC class II (yellow; **C**). Confocal microscopy, original magnification: X40.

Our previous studies have shown that the inflamed corneal stoma is endowed with bone marrow-derived DC and macrophages in addition to keratocytes [3]. We used several antibodies to identify the CCR7^+^ cells and to characterize the cells in the corneal stroma that express this receptor. When double-staining was performed for CCR7 and CD11c in whole mount corneas, we found that CCR7^+^ cells were uniformly CD11c^+^ and mostly located in the peripheral cornea and limbus (Figure 2B). In addition, double-staining for CCR7 and CD11b indicated that the CCR7^+^ cells were also uniformly CD11b^+^ (Figure 2A). This is consistent with our previous findings that the corneal stromal DC were CD11b^+^ and of a monocytic lineage [2,3,25].

To characterize the maturational state of the CCR7^+^ stromal DC, we further performed double-staining for CCR7 and MHC class II. The majority of CCR7^+^ cells were MHC class II positive (Figure 2C). These results indicate that CCR7 is expressed by relatively mature CD11b^+^ and CD11c^+^ DC in the inflamed corneal stoma. In contrast, immature DC in the normal corneas did not express CCR7 (Figure 2F).

### CCR7 is expressed by the ovalbumin-presenting dendritic cells migrating from the cornea to the draining lymph nodes

**Figure 3:**
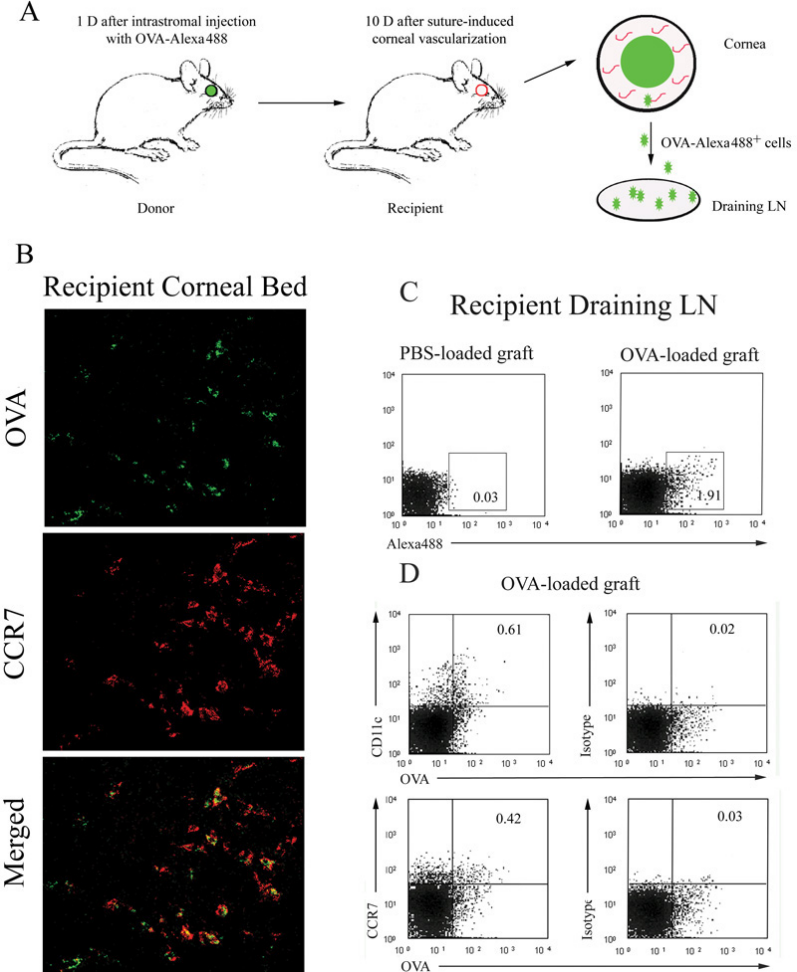
CCR7 was expressed on OVA^+^ cells in both the recipient corneal beds and the draining lymph nodes. OVA-loaded corneal grafts were transplanted into vascularized syngeneic recipient beds (**A**). After 48 h, a portion of CCR7^+^ cells (red) also expressed OVA (green) on their surface in the recipient stromal beds (costained as yellow). Confocal microscopy, original magnification: X40 (**B**). Draining lymph node (LN) cells were harvested and OVA^+^ cells were detected by flow cytometry. Mice with PBS-loaded corneal grafts served as negative controls (**C**). Flow cytometric expression of CD11c and CCR7 on the OVA^+^ cells in the draining LN is shown in **D**.

We assessed the migration of the CCR7^+^ corneal stromal DC in syngeneic OVA-Alexa488-loaded corneal grafts (Figure 3A). Once APC pick up these fluorescence-labeled OVA antigens in the corneal graft and present them on the cell surface, these APC can be detected by their green fluorescence. Forty-eight hours after OVA-loaded corneal grafts were transplanted onto syngeneic lymphatic-rich host corneal beds, we found fluorescence-labeled OVA picked up by CCR7^+^ cells (Figure 3B). These OVA^+^ cells expressed both CCR7 and CD11c on their cell surface as measured by flow cytometry (Figure 3D). These data confirmed that antigen-presenting DC egress from the cornea to the draining LN with persistent expression of CCR7 on their cell surface.

### CCL21 is expressed in the inflamed corneal stroma

**Figure 4:**
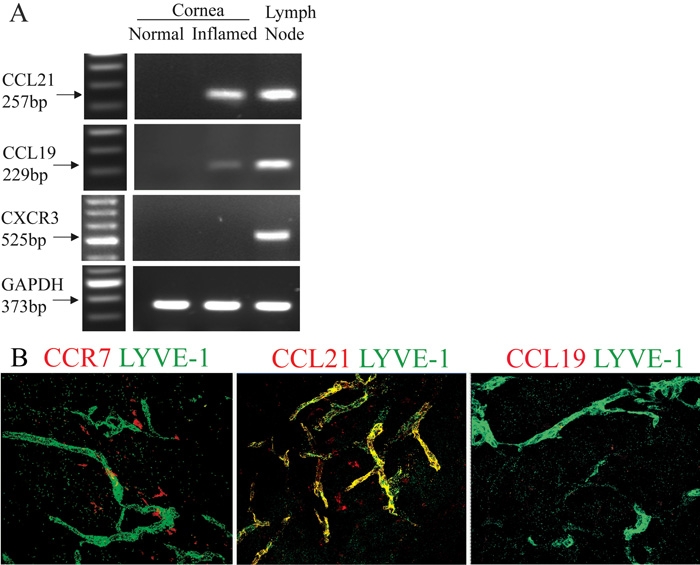
CCR7 and CCL21 were expressed around neo-lymphatic vessels in the vascularized corneas. Transcript of CCL21, but not CCL19 or CXCR3, was significantly expressed in the inflamed corneas. Lymph nodes served as positive control (**A**). In the inflamed corneal stromas, dendritiform CCR7^+^ cells (left, red) and CCL21^+^ cells (middle, red) were near the LYVE-1^+^ lymphatics (green). CCL21 also co-localized with LYVE-1^+^ lymphatics (middle, yellow). No CCL19^+^ cells were detected (right; **B**). Confocal microscopy, original magnification: X40.

We first detected that CCL21 was significantly expressed in the inflamed cornea at mRNA level. However, CCL19 transcript was present only at a low level in the inflamed cornea. Additionally, CXCR3, another receptor for CCL21, was detected neither in the normal nor the inflamed cornea (Figure 4A).

Immunohistochemical studies revealed that CCL21 colocalized with LYVE-1+ (lymphatic vessel endothelial receptor 1) lymphatics in the inflamed corneal stroma. Furthermore, dendritiform CCR7^+^ cells and CCL21^+^ cells were located around LYVE-1^+^ lymphatics. However, no CCL19^+^ cells were detected (Figure 4B).

### CCL21 blockade inhibits corneal dendritic cell trafficking to draining lymph nodes

**Figure 5:**
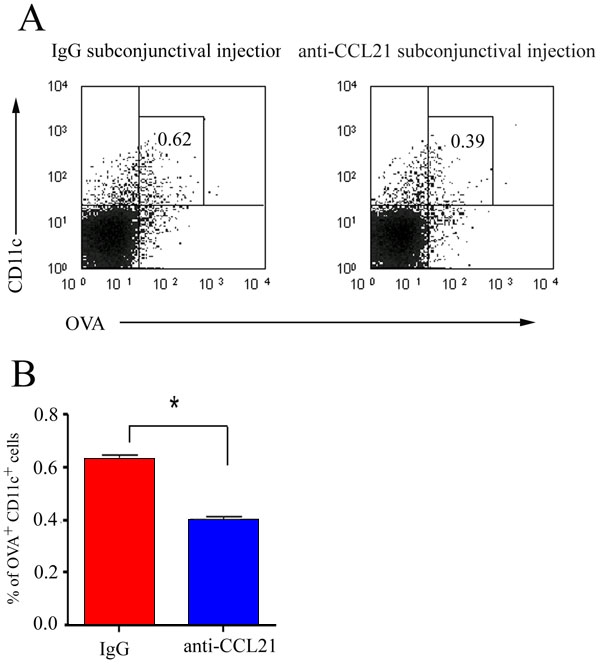
CCL21 blockade inhibited OVA^+^CD11c^+^ dendritic cell trafficking to the draining lymph nodes. Forty-eight h after syngeneic OVA-loaded corneal grafts were transplanted, recipient draining LN cells were harvested from either anti-CCL21 or isotype control IgG-treated groups. The percentage of CD11c^+^ and OVA^+^ cells was analyzed by two-color flow cytometry (**A**). The results are expressed as the mean percentage of CD11c^+^ and OVA^+^ cells±SEM of 3 measurements (the asterisk indicates a p <0.05). The p-value is determined using a two-tailed Student's t-test. The data are representative of three independent experiments (**B**).

To test the hypothesis that corneal DC trafficking is functionally effected by the interaction of CCR7 and CCL21, we blocked local CCL21 function via subconjunctival injection of anti-CCL21. Forty-eight h after transplantion of OVA-loaded corneal grafts to syngeneic host beds, total cells of the draining LN were harvested from either anti-CCL21 or isotype control IgG administration group. Compared with the isotype control IgG group, the group that received subconjunctival anti-CCL21 demonstrated 40% less OVA^+^CD11c^+^ cells in the draining LN (p <0.05; Figure 5).

## Discussion

Investigation of the molecular regulation of DC trafficking is critical for a better understanding of corneal immunity. However, the mechanisms that regulate APC egress from the cornea have not been well understood, although the critical molecular mechanisms that regulate APC infiltration into the inflamed cornea have been much more extensively studied. Similar to other inflamed tissues, the expression of inflammatory cytokines (IL-1 and TNF-α) and inflammatory chemokine receptors (CCR1, CCR2, CCR3, CCR5, and CXCR1) is involved first in recruitment of innate immune cells (e.g., PMNs), and later MHC class II-positive APC (including DC, Langerhans cells, monocytes, and macrophages) from the intravascular compartment and peripheral matrix (limbus) into the cornea, through coordinated upregulation of endothelial cell adhesion factors in the limbal vasculature [28-33]. It is generally accepted that the mobilization of DC from the periphery to the LN is regulated by the gatekeeper receptor CCR7, along with the maturation of DC [11-20]. However, studies of CCR7-mediated DC migration from the cornea to the draining LN are lacking. In this study, we identified CCR7 expression on CD11b^+^ and CD11c^+^ DC in inflamed anterior corneal stroma. In our syngeneic OVA-loaded corneal graft model, both host and graft-derived DC were involved in antigen presentation. CCR7 expression on these DC was found either in the host beds or in the draining LN, indicating persistent CCR7 expression on these antigen-presenting cells during their trafficking. These data suggest that CCR7 expression is highly relevant to DC trafficking from the inflamed cornea to draining LN. Recent data suggest that CCR7 signaling may also be relevant for inhibiting mature DC apoptosis [34], but as of yet we have no data on this for the eye.

Despite studies showing that CCR7 expression typically correlates with the upregulation of MHC and costimulatory molecule expression during DC maturation [9,13,14,35] there are stimuli, such as DNAX-activation protein 12 (DAP12), that can induce CCR7 expression by DC independently of their maturation and acquisition of MHC class II [36,37]. In our study, we found a small portion of MHC class II-negative CCR7^+^ cells, although the majority of CCR7^+^ cells were co-localized with MHC class II in the inflamed cornea. These relatively immature CCR7^+^ DC may have two different fates: (1) they may complete their maturation process under further maturation signals provided by CCL19 and CCL21 [38]; or (2) they may migrate into the draining LN in an immature state. Immature CCR7^+^ DC most likely will be poor in stimulating T cells and hence may induce immune tolerance [39]. Thus, the relationship between CCR7-mediated corneal DC trafficking and immune stimulation or tolerance induction is worthy of further study.

Although the cornea is lymphatic-free, lymphatic vessels can readily grow into it upon inflammatory stimulation [33]. Access of APC to the ipsilateral submandibular LN is facilitated by both these neolymphatic vessels in the corneal matrix and the normally present conjunctival lymphatics [4]. Our finding of CCR7^+^ DC close to CCL21^+^ and LYVE-1^+^ lymphatics suggests that CCL21-CCR7 interactions promote DC access to the lymphoid compartments. Moreover, the inhibitory effect of CCL21 blockade on antigen-bearing DC trafficking strongly supports the hypothesis that CCL21, secreted by lymphatic endothelial cells, promotes CCR7^+^ cell migration into the afferent lymphatics enroute to draining LN.

Another ligand of CCR7 is CCL19, which is expressed by mature DC and stromal cells in the T cell zone of LN [40-43]. Although we only detected CCL19 mRNA (but not protein) expression levels in the inflamed cornea, this does not necessarily refute the contribution of CCL19 to corneal DC trafficking. This ligand may regulate DC trafficking after these cells migrate into the lymphatics or in the LN. However, we were not able to detect this in our experiments, and conclude that the function of CCL19, at least as it pertains to corneal DC trafficking, is not significant.

One of the main challenges in immune modulation is to separate suppression of innate immunity that is critical for immediate host responsiveness to pathogens from T cell-mediated adaptive immunity that plays a critical role in the chronic destructive responses seen in a wide range of conditions, including transplant rejection and various forms of viral keratitis. One strategy that could potentially allow for this differential suppression of adaptive versus innate immunity is to devise interventions that specifically block egress of APC from the cornea to lymphoid reservoirs, but could leave intact the ingress of innate cells into the cornea. Our data here, demonstrating the role of CCR7 in regulating corneal DC trafficking to lymphoid tissues, suggest that CCR7 and CCL21 blockade could potentially offer one such venue for inhibiting the induction of T cell-mediated immunity. Obviously, this approach has more therapeutic benefit than surgical lymphadenectomy, as we described previously [6], to achieve immunologic ignorance. In our studies, however, CCL21 blockade only demonstrated up to 40% inhibitory effect on DC trafficking, similar to the inhibitory effect of CCL21 blockade on skin DC [11], suggesting that CCR7 may not be the exclusive mediator of DC migration from the cornea to lymphoid tissues. Further studies are necessary to determine the precise (overlapping, additive, or synergistic) relationship of VEGFR-3 and CCR7-mediated DC trafficking. Interestingly, CCL21 and VEGF-C, the ligands of CCR7 and VEGFR-3, respectively, are both expressed on lymphatic endothelia, suggesting a close relationship between DC trafficking and lymphangiogenesis in corneal inflammation.
